# Human Case of *Streptococcus suis* Disease, Ontario, Canada

**DOI:** 10.3201/eid2312.171005

**Published:** 2017-12

**Authors:** Jeisa Gomez-Torres, Asim Nimir, James Cluett, Anita Aggarwal, Sameer Elsayed, Deirdre Soares, Sarah Teatero, Yan Chen, Marcelo Gottschalk, Nahuel Fittipaldi

**Affiliations:** Tillsonburg District Memorial Hospital, Tillsonburg, Ontario, Canada (J. Gomez-Torres, A. Nimir);; Tillsonburg Family Physicians, Tillsonburg (J. Cluett);; Woodstock General Hospital, Woodstock, Ontario, Canada (A. Aggarwal, S. Elsayed);; Western University, London, Ontario, Canada (S. Elsayed);; Public Health Ontario Laboratory, Toronto, Ontario, Canada (D. Soares, S. Teatero, Y. Chen, N. Fittipaldi);; University of Montreal, St-Hyacinthe, Quebec, Canada (M. Gottschalk);; University of Toronto, Toronto (N. Fittipaldi)

**Keywords:** *Streptococcus* *suis*, α-hemolytic streptococci, viridans, zoonotes, North America, Canada, Ontario, bacteria, streptococci

## Abstract

We report a case of *Streptococcus suis* human disease in Ontario, Canada, caused by a serotype 2 strain genotypically similar to those commonly isolated from pigs in North America. Initially, the isolate was misidentified as a viridans group *Streptococcu*s. Human *S. suis* infections may be underdiagnosed in North America.

*Streptococcus suis* is a zoonotic agent responsible for both sporadic and outbreak human disease in several Asian countries (*1*–*3*). However, human *S. suis* infections are less frequent in Western countries, and particularly in North America (*3*,*4*). We describe a severe human *S. suis* infection in Ontario, Canada.

The patient, a 69-year-old male farmer, was brought to the emergency department of a rural community hospital in southwestern Ontario after being found unresponsive by his wife. His medical history included congestive heart failure, moderate to severe mitral regurgitation, hypertension, microcytic anemia, and bilateral hearing loss. According to his wife, he had unintentionally lost 9 kg over the previous 2 months and had reported feeling cold the previous evening. He had not reported headache, sore throat, chest pain, cough, shortness of breath, abdominal pain, nausea, vomiting, or diarrhea, and he had not been in contact with persons who were ill.

The patient’s vital signs were normal except for a temperature of 39.9°C. He was comatose and had a Glasgow Coma Scale score of 9. There was no evidence of trauma. Physical examination was unremarkable except for the presence of nystagmus. Hematologic studies showed a leukocyte count of 11.8 × 10^9^ cells/L with 11% neutrophils; platelet count, creatinine, and liver enzymes were all within normal limits. Results of computed tomography of the brain were normal. A chest radiograph revealed moderate bilateral peribronchial thickening with increased interstitial markings. Empiric treatment with intravenous (IV) piperacillin/tazobactam for septic syndrome was initiated. The patient’s neurologic status rapidly deteriorated (Glascow Coma Scale score of 5), and he required intubation. He was transferred to a regional hospital, where empiric antibiotic drugs were changed to IV vancomycin and ceftriaxone. Blood cultures grew a gram-positive α-hemolytic organism, identified by using the Vitek II system as *Streptococcus thoraltensis*, a rare viridans group *Streptococcus*. Treatment was changed to IV penicillin G. After 48 hours, the patient improved, was extubated, and was transferred back to the rural hospital. Upon arrival, he remained confused, was transiently febrile, and had visual hallucinations. IV penicillin G was continued. The patient continued to improve. Repeat blood cultures were negative. Transthoracic 2-dimensional echocardiography did not identify lesions. The patient was discharged on day 10 to complete a 14-day course of IV ceftriaxone at home. He has since fully recovered.

As part of standard procedures, the patient isolate was sent to the Public Health Ontario Laboratory (Toronto, Ontario, Canada), where it was identified as *S. suis* by using matrix-assisted laser desorption/ionization time-of-flight mass spectrometry*.* The genome of the isolate was sequenced by using Illumina Technology (Illumina, San Diego, CA, USA); bioinformatics-based typing (*5*) assigned the isolate to serotype 2 and sequence type (ST) 25. Further phylogenetic analysis determined that the isolates belonged to ST25 clade NAV1 (Figure), which is common among diseased swine in Canada (*6*,*7*) but heretofore not associated with human disease. The isolate, confirmed as serotype 2 by using the coagglutination test (*8*), did not produce the virulence markers muramidase-released protein, extracellular factor, or the hemolysin suilysin.

**Figure:**
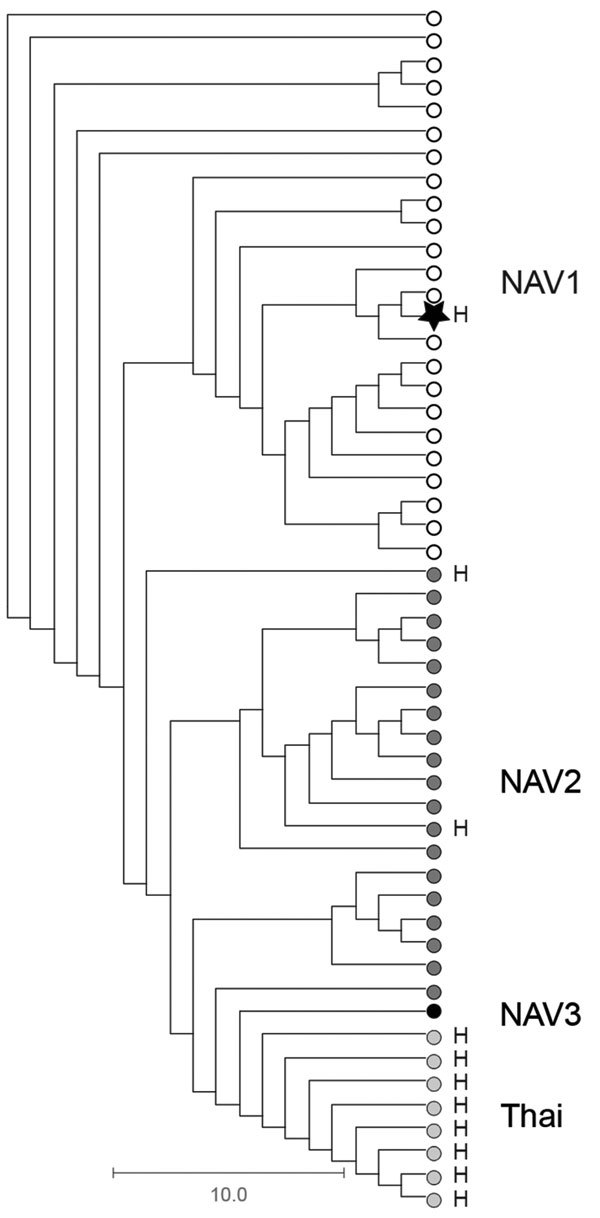
Phylogenetic relationships among *Streptococcus suis* serotype 2 sequence type (ST) 25 isolate from a patient in Ontario, Canada (star), and 51 previously described (*6*) porcine and human serotype 2 ST25 *S. suis* isolates. The cladogram is based on nonredundant single-nucleotide polymorphism loci identified in the genome of all isolates relative to the *S. suis* serotype 2 ST25 core genome, as defined by Athey et al. (*6*). The human isolate from Ontario is genetically more closely related to serotype 2 ST25 strains of clade NAV1 (open circles), which are commonly recovered from diseased pigs in North America and which have not previously been associated with human disease, than to other serotype 2 ST25 clades from North America (NAV2, dark gray circles, and NAV3, black circle) or serotype 2 ST25 organisms from Thailand (light gray circles). Scale bar indicates nucleotide substitutions per site. H, isolates recovered from human infections.

When questioned about swine contact, the patient reported raising ≈2,000 pigs on his farm. There was no evidence of active disease among these animals. However, pigs in porcine farms not deemed high-health status are regularly colonized by *S. suis* (*3*). Most cases of *S. suis* disease in humans have been linked to accidental inoculation through skin injuries (*3*). The patient reported that in the days before his hospitalization, a pig died unexpectedly, and he removed it from the pen without using protective equipment such as gloves or safety glasses. However, there was no indication that this animal had died of a *S. suis* infection. The patient also described transient worsening of his bilateral hearing loss during hospitalization. Hearing loss from *S. suis* infection occurs frequently (*9*).

*S. suis* zoonotic disease has emerged in Asia and occurs frequently in Europe among persons in close contact with pigs (*3*). In contrast, only 8 human *S. suis* cases have been reported in Canada and the United States, which together are the second largest swine producers worldwide (*3*). This lower number of cases may be related to the lower virulence of *S. suis* serotype 2 genotypes circulating in North America (ST25 and ST28) in comparison to serotype 2 genotypes circulating in Europe and Asia (ST1, ST7) (*4*,*6*,*7*). However, *S. suis* infections may be underdiagnosed in North America. Our data and previous reports (*10*) show that the organism is sometimes misidentified as other α-hemolytic streptococci by commercial identification systems. Here, the isolate was initially identified as viridans group *Streptococci*, and only the use of matrix-assisted laser desorption/ionization time-of-flight mass spectrometry at the reference laboratory permitted correct identification. While proper identification was unlikely to have led to a different treatment course in this case, our report underscores the need to increase awareness of *S. suis* as a potential agent of human infections and serves as a reminder to routinely query patients about animal contact, particularly in areas with intensive pig farming operations.

## References

[1] Fulde M, Valentin-Weigand P. Epidemiology and pathogenicity of zoonotic streptococci. Curr Top Microbiol Immunol. 2013;368:49–81. 10.1007/82_2012_277 23192319

[2] Wertheim HF, Nghia HD, Taylor W, Schultsz C. *Streptococcus suis*: an emerging human pathogen. Clin Infect Dis. 2009;48:617–25. 10.1086/596763 19191650

[3] Gottschalk M, Xu J, Calzas C, Segura M. *Streptococcus suis*: a new emerging or an old neglected zoonotic pathogen? Future Microbiol. 2010;5:371–91. 10.2217/fmb.10.2 20210549

[4] Goyette-Desjardins G, Auger JP, Xu J, Segura M, Gottschalk M. *Streptococcus suis*, an important pig pathogen and emerging zoonotic agent-an update on the worldwide distribution based on serotyping and sequence typing. Emerg Microbes Infect. 2014;3:e45. 10.1038/emi.2014.45 26038745 PMC4078792

[5] Athey TB, Teatero S, Lacouture S, Takamatsu D, Gottschalk M, Fittipaldi N. Determining *Streptococcus suis* serotype from short-read whole-genome sequencing data. BMC Microbiol. 2016;16:162. 10.1186/s12866-016-0782-8 27449127 PMC4957933

[6] Athey TB, Teatero S, Takamatsu D, Wasserscheid J, Dewar K, Gottschalk M, et al. Population structure and antimicrobial resistance profiles of *Streptococcus suis* serotype 2 sequence type 25 strains. PLoS One. 2016;11:e0150908. 10.1371/journal.pone.0150908 26954687 PMC4783015

[7] Fittipaldi N, Xu J, Lacouture S, Tharavichitkul P, Osaki M, Sekizaki T, et al. Lineage and virulence of *Streptococcus suis* serotype 2 isolates from North America. Emerg Infect Dis. 2011;17:2239–44. 10.3201/eid1712.110609 22172538 PMC3311171

[8] Gottschalk M, Higgins R, Boudreau M. Use of polyvalent coagglutination reagents for serotyping of *Streptococcus suis.* J Clin Microbiol. 1993;31:2192–4. 8370749 10.1128/jcm.31.8.2192-2194.1993 PMC265720

[9] van Samkar A, Brouwer MC, Schultsz C, van der Ende A, van de Beek D. *Streptococcus suis* meningitis: A systematic review and meta-analysis. PLoS Negl Trop Dis. 2015;9:e0004191. 10.1371/journal.pntd.0004191 26505485 PMC4624688

[10] Fongcom A, Pruksakorn S, Netsirisawan P, Pongprasert R, Onsibud P. *Streptococcus suis* infection: a prospective study in northern Thailand. Southeast Asian J Trop Med Public Health. 2009;40:511–7. 19842437

